# (*E*)-Benzaldehyde (2,4,6-trichloro­phen­yl)hydrazone

**DOI:** 10.1107/S160053681100328X

**Published:** 2011-01-29

**Authors:** Yan-Lan Huang, Deng-Feng Li, Jian Sun, Jin-Hua Gao, Shang Shan

**Affiliations:** a College of Chemical Engineering and Materials Science, Zhejiang University of Technology, People’s Republic of China

## Abstract

The title compound, C_13_H_9_Cl_3_N_2_, was obtained from a condensation reaction of benzaldehyde and 2,4,6-trichloro­phenyl­hydrazine. The mol­ecule assumes an *E* configuration with the phenyl ring and trichloro­phenyl ring located on opposite sides of the C=N bond. The phenyl ring is oriented at a dihedral angle of 42.58 (12)° with respect to the tricholorophenyl ring. In the crystal, the mol­ecules are linked *via* N—H⋯N hydrogen bonds, forming supra­molecular chains running along the *c* axis. π–π stacking is present between parallel trichloro­phenyl rings of adjacent mol­ecules, the face-to-face and centroid–centroid distances being 3.369 (14) and 3.724 (2) Å, respectively.

## Related literature

For the biological activity of phenyl­hydrazone derivatives, see: Okabe *et al.* (1993 ▶). For related structures, see: Shan *et al.* (2003 ▶); Fan *et al.* (2005 ▶); Bolte & Dill (1998 ▶).
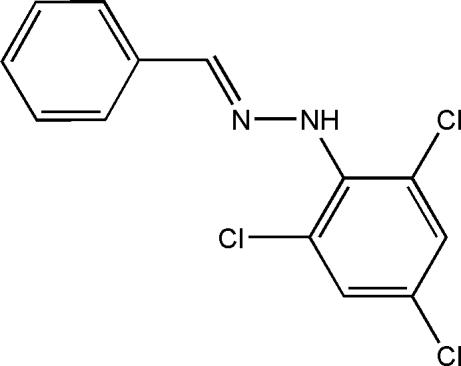


## Experimental

### 

#### Crystal data

C_13_H_9_Cl_3_N_2_
*M* *_r_* = 299.57
Monoclinic, 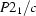

*a* = 13.913 (6) Å
*b* = 12.867 (5) Å
*c* = 7.652 (3) Å
β = 98.739 (5)°
*V* = 1353.9 (9) Å^3^
*Z* = 4
Mo *K*α radiation
μ = 0.66 mm^−1^
*T* = 295 K
0.36 × 0.30 × 0.26 mm

#### Data collection

Rigaku R-AXIS RAPID IP diffractometer
Absorption correction: multi-scan (*ABSCOR*; Higashi, 1995 ▶) *T*_min_ = 0.86, *T*_max_ = 0.92
11730 measured reflections
2436 independent reflections
1936 reflections with *I* > 2σ(*I*)
*R*_int_ = 0.028

#### Refinement

*R*[*F* ^2^ > 2σ(*F* ^2^)] = 0.041
*wR*(*F* ^2^) = 0.105
*S* = 1.06
2436 reflections
163 parameters
H-atom parameters constrained
Δρ_max_ = 0.18 e Å^−3^
Δρ_min_ = −0.32 e Å^−3^

### 

Data collection: *PROCESS-AUTO* (Rigaku, 1998 ▶); cell refinement: *PROCESS-AUTO*; data reduction: *CrystalStructure* (Rigaku/MSC, 2002 ▶); program(s) used to solve structure: *SHELXS97* (Sheldrick, 2008 ▶); program(s) used to refine structure: *SHELXL97* (Sheldrick, 2008 ▶); molecular graphics: *ORTEP-3 for Windows* (Farrugia, 1997 ▶); software used to prepare material for publication: *WinGX* (Farrugia, 1999 ▶).

## Supplementary Material

Crystal structure: contains datablocks I, global. DOI: 10.1107/S160053681100328X/xu5149sup1.cif

Structure factors: contains datablocks I. DOI: 10.1107/S160053681100328X/xu5149Isup2.hkl

Additional supplementary materials:  crystallographic information; 3D view; checkCIF report

## Figures and Tables

**Table 1:** Hydrogen-bond geometry (Å, °)

*D*—H⋯*A*	*D*—H	H⋯*A*	*D*⋯*A*	*D*—H⋯*A*
N1—H1*N*⋯N2^i^	0.95	2.44	3.183 (3)	134

Symmetry code: (i) 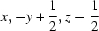.

**Fig. 1.**
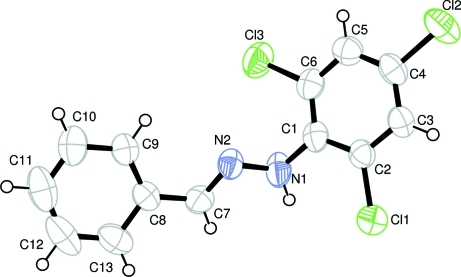
The structure of (I) with 50% probability displacement ellipsoids.

## supplementary crystallographic information

### Comment

As some phenylhydrazone derivatives have been shown to be potential DNA-damaging or mutagenic agents (Okabe *et al.*, 1993), a series of phenylhydrazone derivatives has been synthesized in our laboratory in order to investigate the structure/bioactivity relationship (Shan *et al.* 2003; Fan *et al.* 2005).

The title molecule crystallizes in an E conformation, with the C1-phenyl ring and C8-benzene ring on opposite sides of the C7═N2 double bond. This agrees with the configuration commonly found in phenylhydrazone derivatives (Bolte & Dill, 1998). In the molecule, the phenyl ring is oriented with respect to the tricholorophenyl ring at a dihedral angle of 42.58 (12)°. In the crystal structure, the molecules are linked *via* N—H···N hydrogen bonds to form the supra-molecular chains running along the *c* axis. π-π stacking is present between parallel tricholorophenyl rings of adjacent molecules, the face-to-face distance being 3.369 (14) Å.

### Experimental

2,4,6-Trichlorophenylhydrazine (0.21 g,1 mmol) was dissolved in ethanol (18 ml) and acetic acid (0.3 ml) was added slowly with stirring. The solution was heated at about 333 K for several minutes until it became clear. Benzaldehyde (0.11 g, 1 mmol) was added dropwise with continuous stirring, and the mixture solution was refluxed for 2 h. When the solution cooled to room temperature, microcrystals appeared. The microcrystals were separated from the solution and washed with cold water three times. Recrystallization was performed twice with an absolute ethanol to obtain single crystals of the title compound.

### Refinement

Imino H atom was located a difference Fourier map and refined as riding in as-found relative position. Other H atoms were placed in calculated positions with C—H = 0.93 Å. *U*_iso_(H) = 1.2*U*_eq_(N,C).

### Crystal data

**Table:**

C_13_H_9_Cl_3_N_2_	*F*(000) = 608
*M**_r_* = 299.57	*D*_x_ = 1.470 Mg m^−^^3^
Monoclinic, *P*2_1_/*c*	Mo *K*α radiation, λ = 0.71073 Å
Hall symbol: -P 2ybc	Cell parameters from 4171 reflections
*a* = 13.913 (6) Å	θ = 2.8–26.3°
*b* = 12.867 (5) Å	µ = 0.66 mm^−^^1^
*c* = 7.652 (3) Å	*T* = 295 K
β = 98.739 (5)°	Prism, colorless
*V* = 1353.9 (9) Å^3^	0.36 × 0.30 × 0.26 mm
*Z* = 4	

### Data collection

**Table:**

Rigaku R-AXIS RAPID IP diffractometer	2436 independent reflections
Radiation source: fine-focus sealed tube	1936 reflections with *I* > 2σ(*I*)
graphite	*R*_int_ = 0.028
Detector resolution: 10.0 pixels mm^-1^	θ_max_ = 25.2°, θ_min_ = 3.0°
ω scans	*h* = −16→16
Absorption correction: multi-scan (*ABSCOR*; Higashi, 1995)	*k* = −15→15
*T*_min_ = 0.86, *T*_max_ = 0.92	*l* = −9→9
11730 measured reflections	

### Refinement

**Table:**

Refinement on *F*^2^	Primary atom site location: structure-invariant direct methods
Least-squares matrix: full	Secondary atom site location: difference Fourier map
*R*[*F*^2^ > 2σ(*F*^2^)] = 0.041	Hydrogen site location: inferred from neighbouring sites
*wR*(*F*^2^) = 0.105	H-atom parameters constrained
*S* = 1.06	*w* = 1/[σ^2^(*F*_o_^2^) + (0.0449*P*)^2^ + 0.4002*P*] where *P* = (*F*_o_^2^ + 2*F*_c_^2^)/3
2436 reflections	(Δ/σ)_max_ = 0.001
163 parameters	Δρ_max_ = 0.18 e Å^−^^3^
0 restraints	Δρ_min_ = −0.32 e Å^−^^3^

### Special details

**Table:**

Geometry. All e.s.d.'s (except the e.s.d. in the dihedral angle between two l.s. planes) are estimated using the full covariance matrix. The cell e.s.d.'s are taken into account individually in the estimation of e.s.d.'s in distances, angles and torsion angles; correlations between e.s.d.'s in cell parameters are only used when they are defined by crystal symmetry. An approximate (isotropic) treatment of cell e.s.d.'s is used for estimating e.s.d.'s involving l.s. planes.
Refinement. Refinement of *F*^2^ against ALL reflections. The weighted *R*-factor *wR* and goodness of fit *S* are based on *F*^2^, conventional *R*-factors *R* are based on *F*, with *F* set to zero for negative *F*^2^. The threshold expression of *F*^2^ > σ(*F*^2^) is used only for calculating *R*-factors(gt) *etc*. and is not relevant to the choice of reflections for refinement. *R*-factors based on *F*^2^ are statistically about twice as large as those based on *F*, and *R*- factors based on ALL data will be even larger.

### Fractional atomic coordinates and isotropic or equivalent isotropic displacement parameters (Å^2^)

**Table:**

	*x*	*y*	*z*	*U*_iso_*/*U*_eq_	
Cl1	0.82868 (5)	0.03185 (5)	0.52523 (10)	0.0786 (2)	
Cl2	1.16441 (5)	0.21670 (7)	0.78646 (11)	0.0897 (3)	
Cl3	0.85826 (6)	0.45120 (5)	0.53336 (10)	0.0877 (3)	
N1	0.75114 (15)	0.24687 (17)	0.4714 (3)	0.0746 (6)	
H1N	0.7271	0.1962	0.3849	0.089*	
N2	0.69094 (13)	0.30372 (14)	0.5622 (2)	0.0566 (5)	
C1	0.84849 (16)	0.24097 (18)	0.5442 (3)	0.0557 (6)	
C2	0.89458 (15)	0.14534 (18)	0.5762 (3)	0.0548 (5)	
C3	0.99117 (16)	0.13659 (19)	0.6484 (3)	0.0595 (6)	
H3	1.0203	0.0717	0.6674	0.071*	
C4	1.04329 (16)	0.2259 (2)	0.6915 (3)	0.0612 (6)	
C5	1.00210 (17)	0.3222 (2)	0.6597 (3)	0.0638 (6)	
H5	1.0386	0.3821	0.6883	0.077*	
C6	0.90617 (18)	0.32876 (19)	0.5850 (3)	0.0603 (6)	
C7	0.59985 (17)	0.29350 (17)	0.5147 (3)	0.0582 (6)	
H7	0.5769	0.2464	0.4258	0.070*	
C8	0.53090 (15)	0.35459 (18)	0.5976 (3)	0.0535 (5)	
C9	0.56026 (17)	0.44135 (18)	0.6979 (3)	0.0561 (5)	
H9	0.6254	0.4608	0.7146	0.067*	
C10	0.4942 (2)	0.4996 (2)	0.7737 (3)	0.0760 (7)	
H10	0.5151	0.5578	0.8411	0.091*	
C11	0.3983 (2)	0.4720 (3)	0.7501 (4)	0.0934 (10)	
H11	0.3539	0.5115	0.8011	0.112*	
C12	0.3676 (2)	0.3869 (3)	0.6520 (5)	0.0952 (10)	
H12	0.3022	0.3683	0.6369	0.114*	
C13	0.43269 (18)	0.3272 (2)	0.5741 (4)	0.0758 (7)	
H13	0.4110	0.2694	0.5066	0.091*	

### Atomic displacement parameters (Å^2^)

**Table:**

	*U*^11^	*U*^22^	*U*^33^	*U*^12^	*U*^13^	*U*^23^
Cl1	0.0633 (4)	0.0709 (4)	0.1004 (5)	−0.0018 (3)	0.0087 (4)	−0.0148 (3)
Cl2	0.0506 (4)	0.1110 (6)	0.1040 (6)	−0.0073 (3)	0.0007 (3)	0.0177 (4)
Cl3	0.0985 (5)	0.0642 (4)	0.0989 (5)	0.0182 (4)	0.0100 (4)	0.0040 (4)
N1	0.0620 (12)	0.0951 (16)	0.0601 (12)	0.0270 (11)	−0.0114 (10)	−0.0335 (11)
N2	0.0565 (11)	0.0629 (11)	0.0472 (10)	0.0140 (9)	−0.0023 (9)	−0.0060 (8)
C1	0.0581 (13)	0.0691 (15)	0.0397 (11)	0.0130 (11)	0.0065 (10)	−0.0078 (10)
C2	0.0520 (12)	0.0631 (14)	0.0509 (12)	0.0030 (10)	0.0124 (10)	−0.0055 (10)
C3	0.0505 (13)	0.0658 (14)	0.0639 (14)	0.0065 (11)	0.0144 (11)	0.0089 (11)
C4	0.0470 (12)	0.0816 (17)	0.0564 (13)	0.0007 (11)	0.0131 (11)	0.0108 (12)
C5	0.0634 (15)	0.0684 (15)	0.0606 (14)	−0.0047 (12)	0.0130 (12)	−0.0005 (12)
C6	0.0697 (15)	0.0612 (14)	0.0501 (12)	0.0092 (12)	0.0093 (11)	−0.0009 (11)
C7	0.0608 (14)	0.0596 (13)	0.0492 (12)	0.0031 (11)	−0.0081 (11)	−0.0046 (10)
C8	0.0478 (12)	0.0649 (14)	0.0455 (11)	0.0044 (10)	−0.0002 (9)	0.0120 (10)
C9	0.0554 (13)	0.0670 (14)	0.0447 (11)	0.0102 (11)	0.0039 (10)	0.0081 (11)
C10	0.0832 (19)	0.0897 (18)	0.0567 (15)	0.0249 (15)	0.0159 (14)	0.0066 (13)
C11	0.077 (2)	0.130 (3)	0.081 (2)	0.030 (2)	0.0352 (17)	0.023 (2)
C12	0.0486 (15)	0.136 (3)	0.104 (2)	0.0061 (17)	0.0195 (16)	0.042 (2)
C13	0.0581 (15)	0.0892 (19)	0.0758 (17)	−0.0089 (14)	−0.0034 (13)	0.0176 (14)

### Geometric parameters (Å, °)

**Table:**

Cl1—C2	1.737 (2)	C5—H5	0.9300
Cl2—C4	1.735 (2)	C7—C8	1.458 (3)
Cl3—C6	1.733 (2)	C7—H7	0.9300
N1—N2	1.377 (3)	C8—C9	1.381 (3)
N1—C1	1.386 (3)	C8—C13	1.396 (3)
N1—H1N	0.9526	C9—C10	1.380 (3)
N2—C7	1.271 (3)	C9—H9	0.9300
C1—C2	1.392 (3)	C10—C11	1.367 (4)
C1—C6	1.393 (3)	C10—H10	0.9300
C2—C3	1.378 (3)	C11—C12	1.359 (5)
C3—C4	1.372 (3)	C11—H11	0.9300
C3—H3	0.9300	C12—C13	1.389 (4)
C4—C5	1.372 (3)	C12—H12	0.9300
C5—C6	1.372 (3)	C13—H13	0.9300
			
N2—N1—C1	117.26 (18)	N2—C7—C8	120.9 (2)
N2—N1—H1N	122.5	N2—C7—H7	119.6
C1—N1—H1N	117.4	C8—C7—H7	119.6
C7—N2—N1	117.19 (19)	C9—C8—C13	118.5 (2)
N1—C1—C2	121.0 (2)	C9—C8—C7	121.3 (2)
N1—C1—C6	122.7 (2)	C13—C8—C7	120.3 (2)
C2—C1—C6	116.3 (2)	C10—C9—C8	120.9 (2)
C3—C2—C1	122.5 (2)	C10—C9—H9	119.6
C3—C2—Cl1	118.09 (18)	C8—C9—H9	119.6
C1—C2—Cl1	119.38 (18)	C11—C10—C9	120.2 (3)
C4—C3—C2	118.4 (2)	C11—C10—H10	119.9
C4—C3—H3	120.8	C9—C10—H10	119.9
C2—C3—H3	120.8	C12—C11—C10	120.1 (3)
C3—C4—C5	121.5 (2)	C12—C11—H11	120.0
C3—C4—Cl2	119.22 (19)	C10—C11—H11	120.0
C5—C4—Cl2	119.3 (2)	C11—C12—C13	120.8 (3)
C4—C5—C6	118.9 (2)	C11—C12—H12	119.6
C4—C5—H5	120.6	C13—C12—H12	119.6
C6—C5—H5	120.6	C12—C13—C8	119.7 (3)
C5—C6—C1	122.3 (2)	C12—C13—H13	120.2
C5—C6—Cl3	117.8 (2)	C8—C13—H13	120.2
C1—C6—Cl3	119.84 (18)		

### Hydrogen-bond geometry (Å, °)

**Table:**

*D*—H···*A*	*D*—H	H···*A*	*D*···*A*	*D*—H···*A*
N1—H1N···N2^i^	0.95	2.44	3.183 (3)	134

Symmetry codes: (i) *x*, −*y*+1/2, *z*−1/2.
